# Pericentric inversion of chromosome 6 and male fertility problems

**DOI:** 10.1515/med-2022-0411

**Published:** 2022-01-19

**Authors:** Haitao Fan, Zhe Liu, Peng Zhan, Guoliang Jia

**Affiliations:** Department of Urology, The Second Hospital of Jilin University, Changchun, Jilin Province 130041, China

**Keywords:** male infertility, pericentric inversion, chromosome 6, genetic counseling

## Abstract

As a significant chromosomal structural abnormality, chromosomal inversion is closely related to male infertility. For inversion carriers, the interchromosomal effect explains male infertility, but its specific mechanism remains unclear. Additionally, inversion carriers with different chromosomes have different clinical manifestations. Therefore, genetic counseling is difficult in clinical practice. Herein, four male carriers of pericentric inversion in chromosome 6 have been described. Two patients showed asthenospermia, one showed azoospermia, and the wife of the remaining patient had recurrent miscarriages. Through a literature search, the association between the breakpoint of pericentric inversion in chromosome 6 and male fertility problems are also discussed in this study. Overall, important genes related to asthenospermia in chromosome 6p21 were found, which may be related to the clinical phenotype. These results suggest that physicians should focus on the breakpoints of inversion in genetic counseling.

## 1 Introduction

Male infertility is a common clinical problem reported to reproductive medicine centers. Male factors contribute to approximately 50% of all infertility among couples [1]. Genetic variation is one of the important causes of male infertility. Recently, Tang et al. [2] reported that variants of homozygous helicase for meiosis 1 are responsible for spermatogenic failure. *MLH3* single nucleotide polymorphisms in human populations can lead to male infertility [3,4,5]. The breakpoints of chromosome structural rearrangement could result in additional alterations, such as gene disruption or position effect, which ultimately lead to male infertility [6]. Hence, karyotype analysis is an important component of male infertility analysis [7]. Chromosomal aberrations include structural and numerical abnormalities. Structural abnormalities may affect spermatogenesis and are closely associated with male infertility [8]. Inversion is an important structural aberration and has been proven to be associated with male infertility [8,9,10,11].

The specific mechanisms underlying the effects of inversion chromosome on fertility is still unknown. This is because accurate genetic counseling remains challenging for the inversion carrier. For the carriers with chromosomal structural abnormality, preimplantation genetic diagnosis (PGD) has been recommended to give birth to offspring, as it has been proven to improve live birth rates and decrease miscarriage rates [12,13]. However, recent studies have shown that PGD cannot provide the same prominent benefits for inversion carriers in the Chinese Han population [14]. Moreover, a few cases of familial pericentric inversion have been reported [9,15,16]. Although these inversion carriers have a history of adverse pregnancy, they can still conceive naturally. Natural pregnancy can also be used as an option for inversion carriers [10].

This study reported four males with chromosome 6 inversion. Moreover, the association between pericentric inversion of chromosome 6 and male fertility problems has been discussed considering published cases as well.

## 2 Materials and methods

This study was approved by the Ethics Committee of the Second Hospital, Jilin University. Written informed consent has been obtained from all participants for the publication of these cases.

### 2.1 Patients

The patients included here had visited the andrology outpatient department of the Second Hospital, Jilin University, China. A questionnaire survey was conducted to collect patient data, such as age, marriage status, pregnancy history, genetic family history, anamnesis information, smoking and drinking history, and intervention of drugs. Physical examination was performed to record patients’ height, weight, growth and development information, and testicular size. We included four male carriers with chromosome 6 inversion.

### 2.2 Semen analysis

After abstinence for 3–7 days, patients’ semen was collected in a sterile container and examined by two professional technicians after liquefaction. Semen parameters were detected using the computer-aided semen analysis system (Beion S3, Shanghai Beion Medical Technology Co., Ltd, Shanghai, China). Azoospermia was diagnosed when no sperm was detected in the semen after centrifuging the sample three times and after excluding nonejaculation and retrograde ejaculation. Asthenozoospermia was diagnosed when the percentage of progressive sperm in semen was lower than the reference value of 32%.

### 2.3 Cytogenetic analysis

Peripheral blood (2 mL) was collected from all patients in sterile tubes containing heparin anticoagulant. Lymphocytes were cultured in RPMI-1640 culture medium (including phytohemagglutinin) (Yishengjun; Guangzhou Baidi Biotech, Guangzhou, China) for 72 h. Then, G-banding was performed using standard operating procedure. At least 20 metaphases were analyzed for each patient. The karyotypes were described according to the International System for Human Cytogenetic Nomenclature (ISCN 2016).

### 2.4 Collection of published cases

To explore the relationship between chromosome 6 inversion and male fertility problems, a PubMed search was performed using the keywords “chromosome/inversion/male infertility.” In addition, the database (literature we have read) was analyzed, and cases on chromosomal 6 inversion were collected. We included the cases of chromosomal 6 inversion in adult males; prenatal diagnosis cases were excluded. Moreover, related genes on chromosome 6 were searched using Online Mendelian Inheritance in Man (OMIM; https://www.ncbi.nlm.nih.gov/omim).

## 3 Results

### 3.1 Patient characteristics

**Figure 1:**
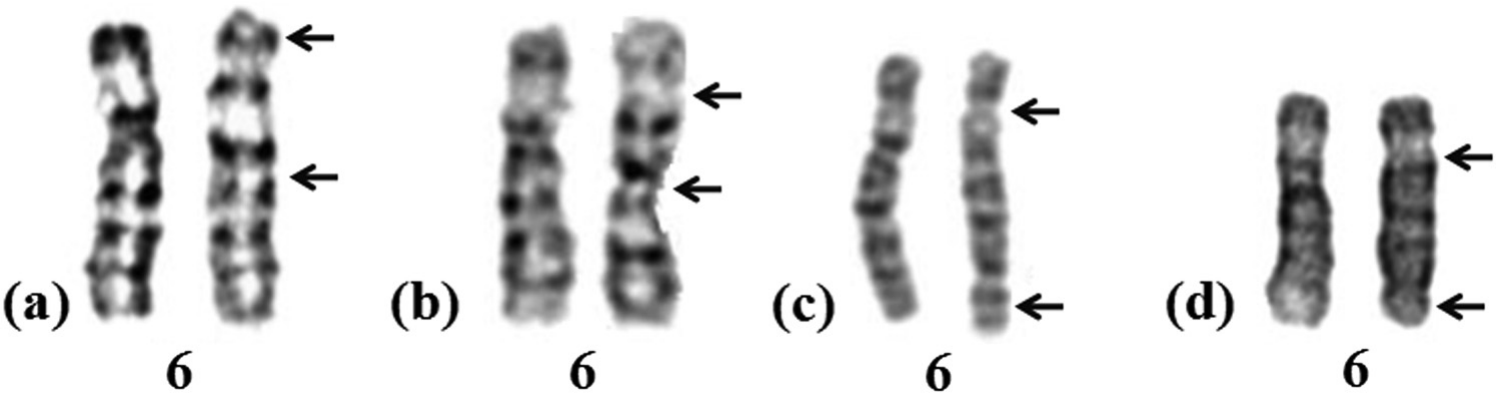
G-banding karyotypes of four patients in this study (a: Case 1; b: case 2; c: Case 3; d: Case 4).

Case 1 involved a 29-year-old man who presented with normal intelligence and phenotype. He visited the hospital for consultation because his wife had two spontaneous miscarriages. Semen analysis showed that semen parameters were within the normal range. Cytogenetic analysis revealed that his karyotype was 46,XY,inv(6)(p23q13) (Figure 1a) and that of his wife was 46,XX. Routine gynecological examination showed that his wife had a normal reproductive function.

Case 2 involved a 25-year-old man who presented to the andrology department because of infertility after 2 years of marriage. Clinical examination showed normal intelligence and phenotype. Semen analysis revealed asthenospermia. Cytogenetic analysis revealed that his karyotype was 46,XY,inv(6)(p21q21) (Figure 1b), whereas that of his wife was 46,XX. Routine gynecological examination showed that his wife had a normal reproductive function.

Case 3 involved a 28-year-old man showing normal intelligence and phenotype who presented to the andrology department because his wife had two spontaneous miscarriages. Semen analysis revealed asthenospermia. Cytogenetic analysis revealed that his karyotype was 46,XY,inv(6)(p21.1q25) (Figure 1c) and that of his wife was 46,XX. Routine gynecological examination showed that his wife’s reproductive function was normal.

Case 4 involved a 33-year-old man who presented to the andrology department because of infertility after 5 years of marriage. He was diagnosed with azoospermia after undergoing semen analysis twice. Cytogenetic analysis revealed that his karyotype was 46,XY,inv(6)(p11q25) (Figure 1d), whereas that of his wife was 46,XX. Routine gynecological examination showed that his wife had a normal reproductive function.

### 3.2 Review of the literature

**Figure 2:**
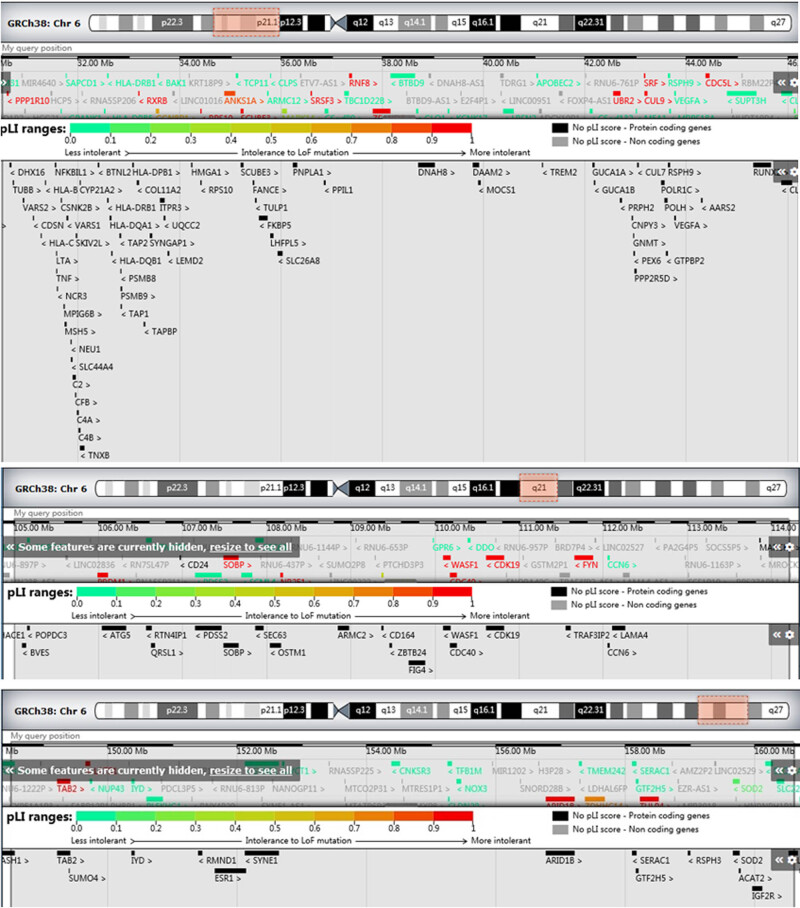
Genes associated with chromosomes 6p21, 6q21, and 6q25 in DECIPHER.

A total of ten carriers involving chromosomal 6 inversion were found in literature. Karyotype and clinical findings of these cases were collected and are summarized in Table 1. Including the four cases in our study, a total of seven cases of male infertility (including azoospermia, oligozoospermia, and asthenospermia) were identified. Four cases were associated with recurrent miscarriage, whereas the other three showed normal fertility. To analyze the possible causes of male infertility, we searched the related pathogenic genes at chromosomes 6p21, 6q21, and 6q25 using DECIPHER (https://www.deciphergenomics.org/; Figure 2). Meanwhile, genes associated with sperm function were searched using OMIM, and information on the genes and their loci and functions were collected. We identified six important genes at 6p21, 6q15, 6q21, and 6q25 that are related to spermatogenesis or sperm function (Table 2).

**Table 1:** Clinical findings in the couples’ with a male partners carrying chromosome 6 inversion

Cases	Karyotype	Family history	Clinical findings	Reference
1	inv(6)(p21q21)	N/A	Obstructed azoospermia	[25]
2	inv(6) (p23q25)	N/A	Recurrent miscarriage	[26]
3	inv(6)(p22q22)	N/A	Oligozoospermia	[27]
4	inv(6)(p23q23)	Pericentric inversions with observed recombinants in offspring	Normal fertility	[28]
5	inv(6)(p21q27)	Pericentric inversions with observed recombinants in offspring	Normal fertility	[28]
6	inv(6)(p22q24)	N/A	Recurrent abortion	[29]
7	inv(6)(p23q23.3)	N/A	Infertility	[30]
8	inv(6)(p21.3q25)	Daughter carrying inv(6)(p21.3q25)	Normal fertility	[31]
9	inv(6)(p22q24)	N/A	Recurrent miscarriage	[32]
10	inv(6)(p12q21)	N/A	Infertility	[33]
11	inv(6)(p23q13)	N/A	Recurrent miscarriage	This study
12	inv(6)(p21q21)	N/A	Asthenospermia	This study
13	inv(6)(p21.1q25)	N/A	Asthenospermia	This study
14	inv(6)(p11q25)	N/A	Azoospermia	This study

N/A, not applicable.

**Table 2:** Breakpoints and genes related to sperm function on chromosome 6

Breakpoint	Gene	Description/Phenotype	Gene function
6p21	*SLC26A8*	Spermatogenic failure-3	The mutations in the gene associated with severe asthenozoospermia
	*TCTE1*	T complex-associated testis-expressed 1	*TCTE1* knockout mice are sterile due to asthenozoospermia
	*DNAH8*	Spermatogenic failure-46	Spermatogenic failure-46 (SPGF46) is characterized by male infertility due to asthenoteratozoospermia
6q15	*SPACA1*	Sperm acrosome-associated protein 1	SPACA1 is a transmembrane protein that localizes to the equatorial segment of spermatozoa and appears to function in sperm–egg fusion
6q21	*ARMC2*	Spermatogenic failure-38	Spermatogenic failure-38 is characterized by primary infertility and asthenoteratozoospermia due to multiple morphologic abnormalities of the flagella
6q25	*ESR1*	Estrogen receptor 1	ESRA/ESRB knockout mice were infertile. They exhibited various stages of spermatogenesis, but the numbers and motility of epididymal sperm were reduced significantly

## 4 Discussion

Chromosomal inversion is a significant chromosomal structural abnormality. Male inversion carriers may be infertile because of spermatozoa production with unbalanced chromosome [17]. Another possible reason for male infertility is that DNA fragmentation is higher in sperm with unbalanced chromosomal content [18]. Although inversion carriers are potentially infertile, patients with normal fertility have often been observed in clinical practice. Therefore, genetic counseling remains a challenge for inversion carriers. This study reported four males showing pericentric inversion in chromosome 6. Two of these had asthenospermia, one had azoospermia, and the wife of the last patient had recurrent miscarriages.

Genetic counseling varies for inversion carriers showing different clinical phenotypes. For inversion carriers with recurrent spontaneous abortion, the couples can choose PGD to reduce the abortion rate and increase the chances of pregnancy [13]. Moreover, these carriers can opt for natural pregnancy along with prenatal diagnosis [10]. For inversion carriers with asthenospermia, PGD treatment should be recommended [19]. For inversion carriers with azoospermia, the possible etiology needs further analysis.

Through a literature search, the relationship between the breakpoint of chromosome 6 inversion and male infertility has been further reviewed. A total of seven patients showed infertility, azoospermia, oligozoospermia, or asthenospermia. The genomic region of the chromosome 6 is far too big. We searched the related pathogenic genes at chromosomes 6p21, 6q21, and 6q25 using DECIPHER, and found more than 60 important pathogenic genes on chromosome 6p21. To further explore the genes related to spermatogenesis, six important genes were identified at the breakpoint of chromosomal 6 inversion. The solute carrier family 26, member 8, T-complex-associated testes-expressed, and Dynein, axonemal, and heavy chain 8 genes are located on chromosome 6p21 and are associated with severe asthenozoospermia [20–22]. The carriers of inv(6)(p21q21) and inv(6)(p21.1q25) presented with asthenospermia, which may be related to the interference of these gene structures. The armadillo repeat-containing protein 2 gene mapped on chromosome 6 at 6q21 is associated with primary infertility and asthenoteratozoospermia [23]. The carrier of inv(6)(p12q21) (case 10 in Table 1) showed infertility, which may be related to the functional change of these genes. The estrogen receptor 1 gene located on chromosome 6q25 could influence spermatogenesis. ESRA/ESRB knockout mice were found to be infertile [24]. The carrier of inv(6)(p11q25) in this study showed azoospermia, which may be related to the gene change, and can be used for further analysis.

## 5 Conclusion

We report four male carriers of chromosome 6 inversion. Important genes associated with asthenospermia in chromosome 6p21 were found; these may be related to the clinical phenotype of these patients. Considered together with the published literature, these results suggest that physicians should focus on the breakpoints of inversion in genetic counseling.
